# Regulation of aquaporins in plants under stress

**DOI:** 10.1186/s40659-018-0152-0

**Published:** 2018-01-16

**Authors:** Ranganathan Kapilan, Maryam Vaziri, Janusz J. Zwiazek

**Affiliations:** 1 Department of Botany, University of Jaffna, Jaffna, Sri Lanka; 2 Department of Renewable Resources, University of Alberta, Edmonton, AB Canada

**Keywords:** Aquaporin, Gating, Gene regulation, Environmental stresses, Phosphorylation, Water transport

## Abstract

Aquaporins (AQP) are channel proteins belonging to the Major Intrinsic Protein (MIP) superfamily that play an important role in plant water relations. The main role of aquaporins in plants is transport of water and other small neutral molecules across cellular biological membranes. AQPs have remarkable features to provide an efficient and often, specific water flow and enable them to transport water into and out of the cells along the water potential gradient. Plant AQPs are classified into five main subfamilies including the plasma membrane intrinsic proteins (PIPs), tonoplast intrinsic proteins (TIPs), nodulin 26 like intrinsic proteins (NIPs), small basic intrinsic proteins (SIPs) and X intrinsic proteins (XIPs). AQPs are localized in the cell membranes and are found in all living cells. However, most of the AQPs that have been described in plants are localized to the tonoplast and plasma membranes. Regulation of AQP activity and gene expression, are also considered as a part of the adaptation mechanisms to stress conditions and rely on complex processes and signaling pathways as well as complex transcriptional, translational and posttranscriptional factors. Gating of AQPs through different mechanisms, such as phosphorylation, tetramerization, pH, cations, reactive oxygen species, phytohormones and other chemical agents, may play a key role in plant responses to environmental stresses by maintaining the uptake and movement of water in the plant body.

## Background

### Structure and function of plant aquaporins

Aquaporins (AQPs) are transmembrane proteins, which form channels in intracellular and plasma membranes to facilitate rapid movement of water in either direction [1]. Water molecules can move toward the center of the channel by the protein’s electrostatic forces and flow across the water channel pore in both directions down its potential gradient [2]. In addition to water, some major intrinsic protein (MIP) family members can also transport glycerol, CO_2_, urea, ammonia, hydrogen peroxide, boron, silicon, arsenite, antimonite, lactic acid [3, 4] and O_2_ [5]. The molecular weight of AQP family members ranges from 23 to 31 kDa [4].

**Fig. 1:**
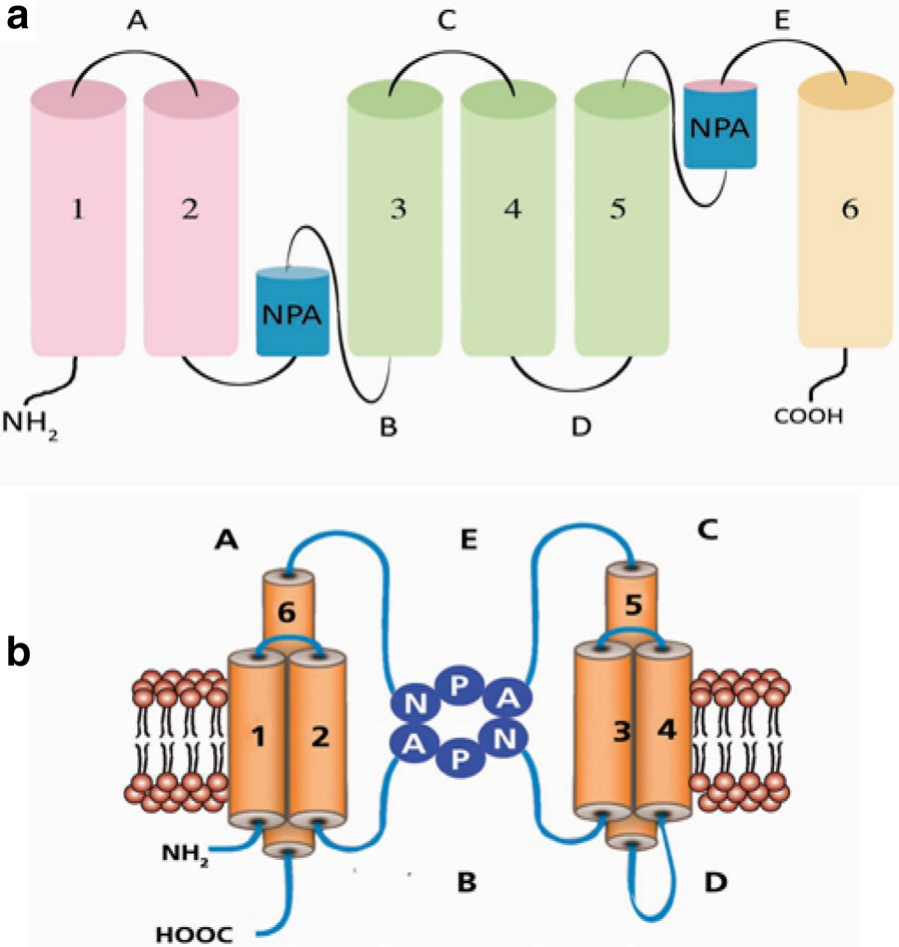
**a** Protein arrangement showing how the two regions of helical domains interact to form the three dimensional structure of the protein. Pore of the AQP is composed of two halves called as hemipores. MIPs consist of six transmembrane domains connected by five loops (A–E), with cytoplasmic N- and C-termini. Locations of NPA (Asn-Pro-Ala) motifs are at the loops B and E. **b** Functional AQP formed by the interaction of the two hemipores [30, 31]

All major intrinsic proteins, including AQPs, consist of six transmembrane helices with N and C termini facing the cytosol. These structures are considered to be helical domains that are packed together (Fig. 1). As part of their structure, they also have five loops (A–E) joining to the transmembrane helices. Loop B and D are intracytoplasmic and A, C and E are extracytoplasmic [6, 7]. The N terminus of each protein is located on the cytoplasmic side of the membrane. Four AQP monomers assemble to form tetrameric holoproteins. Tetramers are stabilized by hydrogen bonds and interactions among the monomer loops [6]. Two conserved loops (B and E) are extremely hydrophobic and contain an internal repeat of Asparagine-Proline-Alanine residues that form NPA motif, which is extended into the pore from both sides of the membrane. This motif is among the most important features in all AQPs to maintain their function. The hydrophobic NPA motif is located at the first intracellular (loop B) and the third extracellular loops (loop E) and form short helices. Loop C also connects to the loop B and E. This connection is functionally necessary for water permeability. These helices fold back into the membrane from the opposite directions [6]. The appearance of AQP protein can be compared with an hourglass and the two hemipores facing each other in reverse within the membrane of AQP [8].

The principle function of AQPs in plants is regulation of transmembrane water transport in situations where water flow needs to be adjusted or the flow is critically low [9]. In plants, AQPs are present in almost all organs including roots, leaves, stems, flowers, fruits, and seeds [10, 11]. Change in the hydraulic conductivity of a plant could be partially controlled by the AQP activity, especially by PIPs [12, 13]. Studies also suggest that AQPs can control water flow across cell membranes either by the change in the AQP abundance or by the change in the water flow rate [3]. The function of the AQPs can be influenced by their interaction with other physiological and biochemical processes of the plant [14].

### Cellular and subcellular localization of aquaporins

As indicated above, the majority of AQPs present in plants are located in either the tonoplast or the plasma membrane and some are possibly localized in the endomembranes. Sequence analyses suggest that it is possible that the level of phosphorylation of the AQP subunits determines their subcellular localization in the plasma membrane or in intracellular vesicles [15]. AQPs can also be found in the plasmalemmasomes and inside of the plasma membrane that extends into the vacuole. The presence of AQPs in the plasmalemmasomes suggests that they may play a significant role in the osmotic balance and turgor maintenance of mesophyll cells [16].

### The plant aquaporin family

Different plant AQPs have different substrate specificities, localization, as well as transcriptional and posttranslational regulations [17, 18]. Depending on membrane localization and amino acid sequence, AQPs in higher plants are classified into five subfamilies [19] including plasma membrane intrinsic proteins (PIPs), tonoplast intrinsic proteins (TIPs), nodulin-26 like intrinsic proteins (NIPs), small basic intrinsic proteins (SIPs), and X intrinsic proteins/uncharacterized-intrinsic proteins (XIPs) [20–22]. Plant species typically have a higher number of AQP genes than animals, ranging from 30 to 70 [4, 18, 23]. For example, in poplar, 55 AQP genes have been identified [22, 24].

### Plasma membrane intrinsic proteins (PIPs)

The plasma membrane intrinsic proteins constitute the largest plant AQP subfamily with the molecular weight of around 30 kDa and an isoelectric point of 9.0. The majority of PIPs have been identified in the plasma membranes and they are generally localized in organs characterized by large fluxes of water i.e. vascular tissues, guard cells and flowers. They have several basic amino acids at the C-terminal. Among the 35 full-length aquaporin genes of the *Arabidopsis* genome, 13 encode for PIPs [22].

Based on the sequence similarities, PIPs are classified into two subgroups, PIP1s and PIP2s. The differences between these two subgroups is the length of amino and C-termini, amino acid substitutions, and their water permeability. Different isoforms of PIP1s and PIP2s are generally localized in almost all parts of the plant including roots and leaves, and the morphology and physiological functions of these organs can be totally different with regard to water or CO_2_ transport [25]. Based on the research and phylogenetic analysis of *Populus trichocarpa*, among the 55 full-length MIP protein sequences, there are 15 active PIPs (five PIP1s and ten PIP2s) [21, 22].

### Pip1s

Despite the fact that the amino acid sequences are similar in both PIP1 and PIP2 subgroups, their water transport ability and cellular functions are very different. The PIP1 subgroup has five members (PIP1;1 to PIP1;5), whereas the PIP2 subgroup has eight isoforms (PIP2;1–PIP2;8) [1]. PIP1 proteins have longer N-terminal but shorter C-terminal tails compared with PIP2. PIP1 proteins are usually present in the plasma membrane [26] and are considered to have low water permeability [27]. Some of the PIP1 proteins are not able to act independently and they must form heterotetramers with the PIP2 monomers to be able to facilitate water permeability [28], however, there is also strong evidence demonstrating their importance in regulating water permeability. In tobacco (*Nicotiana tabacum*) plants, reducing the expression of NtAQP1, a member of the PIP1 family, caused a decline of root hydraulic conductivity and decreased resistance of plants to water stress. In pea (*Pisum sativum*), PIP1 was demonstrated to play an important role in water absorption during seed water uptake [25]. Some members of the PIP1 subgroup are also considered to be responsible for transporting glycerol and CO_2_ in addition to water [29].

### Pip2s

Aquaporins of the PIP2 subfamily are thought to be more efficient as water channels than members of the PIP1 group and different isoforms of PIP2 are considered to be the main pathway for cell-to-cell water transport [25]. Different studies using *Xenopus oocytes* showed that overexpression of the PIP2s significantly increased the water permeability compared to the control group. Compared with PIP1s, PIP2 AQPs have a shorter N-terminal and a longer C-terminal ends with an additional stretch of 4–10 amino acids located in the first extracytosolic loop. These features can contribute to cell-to-cell water transport in roots, leaves, reproductive organs, and seeds.

### Tonoplast intrinsic proteins

Tonoplast Intrinsic Proteins (TIPs) are the most abundant AQPs in the vacuolar membrane [18], and the exact role of TIPs in the cellular membranes of different plant species is known. TIPs were the first proteins with water-transporting function that have been identified in vacuolar membranes of *Arabidopsis thaliana* [32]. TIPs possess molecular weight of between 25 and 28 kDa and an isoelectric point of about 6.0 [33]. In maize, *Arabidopsis* and rice, the TIP group consists of five subgroups with respect to their sequence homologies: TIP1, TIP2, TIP3, TIP4 and TIP5. TIPs are predominantly located in the tonoplast, but some TIP isoforms are found in the membrane surrounding the protein storage vacuoles and small vacuoles [34]. Phylogenetic analysis of *Populus trichocarpa* has revealed that there are 17 TIPs present among the 55 full-length MIP protein sequences [22].

Plant vacuoles are generally considered as a cellular storage compartment, but they can have many other functions in addition to storage. Vacuoles may contain hydrolytic enzymes and secondary metabolites such as alkaloids or storage proteins, especially in seeds. Vacuolar membranes (tonoplasts) are involved in maintaining water and osmotic balance and function as a barrier to intracellular transport. In addition to intracellular water movement and non-limiting water flow through the membranes, TIPs are considered to play an important role in transporting small solutes and gases [35].

Water permeability of the tonoplast is considered to be much higher than that of the plasma membrane, because of the abundance of AQPs in the tonoplast [34] and this leads to rapid osmotic adjustment of the cytoplasm and maintenance of cell turgor pressure. In addition to TIP biological roles, these proteins can also be used as vacuolar markers. Using specific antibodies, it was demonstrated that different TIP isoforms can have different physiological functions. For example, in ice plant (*Mesembryanthemum crystallinum*) the abundance of TIP1; 2 decreased when the plant was exposed to salt stress. This suggests that TIPs may play a role in stress responses [36].

### Nodulin intrinsic proteins (NIPs)

Plants of the Leguminosae family can be colonized by nitrogen fixing bacteria and their roots form nitrogen-fixing nodules. The formation of nodules is the result of symbiotic relationship between the plant and bacteria. During the formation of nodules, nodulin proteins are expressed by plants and transferred to the membranes. Most of the NIP proteins have similar sequences to the nodulin-26 protein that is expressed at the symbiosome membrane (SM) when the root of leguminous plants is infected by rhizobacteria. Nodulin 26 (Nod26) is considered to be a major integral protein of the symbiosome membrane. NIPs form about 10% of total symbiosome membrane protein [37].

It has been shown that in root nodules, NIPs play an important role in transporting water between the bacteria and the host plant. NIPs are different from other plant MIPs and located in plasma and intracellular membranes of both leguminous and non-leguminous plants [1]. Although, Nod26 and other NIPs play a similar role as transporters of water and small solutes, compared to other aquaporins, NIPs have lower water permeability. In poplar, *Populus trichocarpa*, there are eleven NIPs among the full length MIP protein sequences [21, 22]. NIPs also play an important role in drought and salt stress responses and in maintaining plant water balance. Under stress conditions, phosphorylation of NIPs increased, indicating that, similarly to other AQPs, these proteins may play a key role in plant stress responses [38].

### Small basic intrinsic protein (SIPs)

The SIP subfamily is not structurally and functionally well characterized. Proteins of this new subfamily are small, like TIPs, but compared to TIPs, they are very basic. The main reason for their small size is a very short cytosolic N-terminal region compared to the other plant AQPs. In cotton, SIPs are categorized into one SIP1 subgroup. Based on the NPA sequence, SIP1 is divided into the SIP1; 1 and the SIP1; 2 [25]. Compared to other AQP subfamilies, SIPs have shown completely different sites of characteristic residues, which can suggest that different SIP isoforms may have different solute permeabilities. It was observed that Alanine residue present in the first NPA motif was converted to Tyrosine in the first four SIPs of cotton. The Asparagine-N residue present in the first NPA motif of SIP1; 1 was converted to Asparagine-d. These changes in the amino acids (Asparagine-*N* to other amino acids) are among the important types of the conversion of Asparagine-*N* in the NPA motif among the aquaporins found in cotton [23].

### X intrinsic proteins

The first characterization of this newly discovered subfamliy was carried out in upland cotton [23]. XIPs have been characterized in protozoa, fungi, mosses, and dicots. There have been 19 XIP members described including 5 XIPs in *Populus*. The remaining ten were described from dicots other than *Populus*, three from moss and one was from a protozoa. XIP homologs were absent from monocots [17]. Expression analysis of *Populus* MIPs indicates that *Populus* XIPs do not show any tissue-specific transcript abundance. However, XIP subfamily is not functionally well characterized in poplar plants [22]. In grapevine, VvXIP1 was found to play a role in osmotic regulation in addition to H_2_O_2_ transport and metal homeostasis [39].

### Regulation of aquaporin gene expression

Gene expression is a process through which gene information is converted into proteins. The first evidence concerning the role of AQPs in plants came from the studies of their gene expression in different cells, tissues and organs after exposure to different environmental conditions. The abundance of AQPs is critical for understanding their function. Expression of AQPs may be altered by abiotic factors including drought, salinity, low temperatures, and wounding. AQP expression is also affected by phytohormones including abscisic acid and gibberellins [40, 41]. Since *PatPIP1* from a *Populus tremula* × *P. alba* clone was strongly expressed in response to salinity stress, it can be assumed that expression of AQPs is an important mechanism in plant responses to salt-induced water stress [42]. There are several approaches for studying and analyzing gene expression of protein channels in plants. Reverse transcription quantitative PCR (RT-PCR) technique is one of the most common techniques for measuring the mRNA abundance, however, it may be cumbersome since it requires the right design of experimental conditions such as specific and efficient primers [43].

Regulation of gene expression is a stress response mechanism where a gene is transcribed, mRNA is translated into a protein and the protein is correctly targeted. The regulation of water transport through AQPs is complex and involves different mechanisms and factors. MIP gene expression is regulated in a cell-specific manner via hormones and by environmental factors such as water stress, pathogens, low temperature and salinity. MIP gene expression can be regulated by many signaling pathways and through complicated transcriptional, translational and posttranscriptional controls and it is difficult to distinguish a standard expression pattern for each AQP gene [11, 44]. In plants, regulation of AQP expression has been used to evaluate the protein’s activity in the osmotic water permeability and how the manipulation of the expression of the same genes acts. Different studies suggest that low abundance of AQP proteins reduces the water permeability and the high abundance (over-expression) increases the hydraulic conductivity of biological membranes [45]. AQP gene expression can be regulated through different processes such as methylation, phosphorylation, heteromerization and protonation. These mechanisms have been demonstrated to affect AQP trafficking through the secretary pathway to reach the plasma membrane or opening and closing of the pores. Following AQP gene expression patterns in different plant species and specific tissues can also help identify their roles in responses to environmental stresses.

### Gating through phosphorylation and dephosphorylation

Plant responses to changes in water availability require rapid regulation of membrane water permeability. AQP gating involves opening and closing of the AQP water channel pore and phosphorylation and dephosphorylation of AQPs are considered to be important mechanisms regulating their activity [46]. Gating can be also regulated by protons (H^+^) and divalent cations [47]. The enzymes involved in gating are protein kinases and phosphatases. Phosphorylation enhances AQP activity [9] by keeping the AQP pores in the open state [48]. It has been shown using different approaches such as mass spectrometry analyses and the antibodies for phosphorylated AQP peptides that there are different sites of phosphorylation in AQPs of the PIP, TIP and NIP subfamilies.

Phosphorylation of plant AQPs can be directly involved in rapid channel gating, protein trafficking and opening and closing of the pore. It has been confirmed that the loop B and the N- and C-terminal tails of AQPs are the important sites in water channel regulation. A conserved phosphorylation site has been determined in the loop B of almost all plant PIPs [49]. In vivo and in vitro phosphorylation of serine residues within the N- and C-termini has been reported for some plant species. In addition to the phosphorylation of N- or C-terminal serine, a Serine residue located in the cytoplasmic loop close to the first NPA motif can also be phosphorylated. The serine residue is conserved in all plant PIPs and in some TIPs, and remains within protein kinase phosphorylation sequence (Arg/Xaa-Lys-Xaa-Ser-Xaa–Xaa-Arg) recognized by several protein kinases, such as calcium-dependent protein kinases [46].

*Xenopus laevis* oocytes have been used as a reliable and convenient system for understanding the function and regulation of AQPs. The role of phosphorylation in aquaporin activity has been investigated using kinases and phosphatases. Adding a cAMP antagonist, the water transport activity of PvTIP3;1 in *Xenopus* was increased, however, mutation in the phosphorylation site could prevent this effect [1].

### Regulation of AQPs by tetramerization

AQPs are capable of forming homotetramers and heterotetramers in the biological membranes [50]. Forming homo- or hetero-tetramer structures enables AQPs to be trafficked to cellular membranes and to function as channel transporters. Among the different PIP isoforms, PIP1 subgroup has greater water permeability compared to PIP2 isoforms [27]. However, PIP2 proteins may interact with PIP1 members by forming heterooligomer structures that can function as water channels [50, 51]. Expressing PIP1s in conjunction with PIP2s can significantly improve the water permeability [48, 52]. ZmPIP2s in maize have been shown to be functional only if they interact with ZmPIP1;2 and the membrane water permeability is significantly increased when they are co-expressed with ZmPIP2 in *Xenopus* oocytes [7, 50]. Also, SoPIP1 in spinach (*Spinacia oleracea*) has been shown to have low water permeability whereas its heterotetramer with PIP2 had relatively high water permeability [48, 52]. Although heterotetramers usually provide greater stability and folding of the proteins resulting in highly efficient water transport across biological membranes, not all of the AQP subgroups are activated by forming heterotetramers [50].

The lack of expression clearly shows that heterotetramers may not be an important factor for the efficient water transport in all plant tissues, for example, *Zea ma*ys *Zm*PIP2;5 was lacking in shoot tissues of maize [27], and *Nt*PIP2 was lacking in tobacco stigmas [53]. In fact, some PIP2s, TIPs and NIPs are functional when they are expressed alone in heterologous conditions. In some plants, PIP1s are not able to form tetramers individually, but when PIP1s are co-expressed with PIP2s, then only the PIP1s become active water channels and this proves that forming heterotetramer structures can be an important factor in activating or regulating plant AQPs. Generally, Loop E of AQPs is considered to control tetramer formation in plants [50].

### Regulation of aquaporins by plant hormones

Plant hormones can play an important role in AQP regulation. Expression of some plant AQPs is regulated by plant hormones such as gibberellins [40–42], abscisic acid (ABA) [54–56], cytokinins and auxins [41]. Interaction between gibberellins and brassinosteroids has been shown to control AQP expression [42, 56]. ABA can also regulate plant AQP function and induce stomatal closure. Treatments with exogenous ABA can alter the root hydraulic conductivity [57, 58]. ABA also controls the transcription of many AQP isoforms in different parts of plant species [55]). It also regulates transcription of *PIP1* gees of guard cells in maize [42, 56]. Applying ABA also increased the abundance of AtAQPs in *Arabidopsis* [54].

TIPs can also be regulated by plant hormones. In rice, the application of gibberellic acid and ABA increased the expression of *OsAQP* gene, responsible for encoding the TIP in leaves and roots [40]. In ginseng (*Panax ginseng*) cells, PgTIPI was slightly more abundant when the cells were treated with exogenous cytokinins and less abundant when exogenous auxins were supplied [41]. Indole-3-acetic acid (IAA) has been reported to reduce the hydraulic conductivity of root cortical cells in *Populus alba* ×  *P. tremula* var. *glandulosa* [42]. Studies in *Arabidopsis* have shown that IAA acts through the auxin Response Factor 7 (ARF7) and reduces the expression of most PIPs at both transcriptional and translational levels. Salicylic acid, a hormone produced in response to pathogens and abiotic stresses, has been shown to similarly respond to salt stress and decreased the abundance of PIPs and root hydraulic conductivity by a ROS-mediated mechanism. Other growth inducing hormones such as gibberellic acid (GA3) and ethylene have also been reported to regulate AQP expression [42, 59, 60], but the mechanisms contributing to this response are little known.

### Effect of pH and cations on aquaporin activity

The effects of pH on regulation of AQP activity is not well known, however, several studies suggest that AQPs pose structural features which can act as pH sensors [61]. In addition, the position of histidine plays a key role in AQP regulation by pH [62, 63]. Regulation of PIPs by pH is very important during anoxic stress in plants. The pH change in the cytosol can be recognized by the histidine (His-197) residue in the intercellular loop [61]. Reversible reduction of AQP function caused by high concentrations of H^+^ was observed in many plants [64]. In spinach, under flooding condition, the cytosolic pH is dropped and the histidine residue (His193 in SoPIP2.1) is protonated [48]. In *Arabidopsis* under low cytosolic pH, a conserved H (His)-197 residue of the loop D is protonated and subsequently, the water flow can be reduced due to the closure of pores [61]. It was recently proposed that PIP2 phosphorylation of the loop B serine residue is triggered by a stimulus that is likely linked to water status at the cell level. If PIP2 is dephosphorylated in this residue, pH regulation becomes a significant regulatory mechanism for water transport [65]. The trembling aspen (*Populus tremuloides*) that were inoculated with *H*. *crustuliniforme* had a maximum root hydraulic conductivity at pH 7 than the non inoculated plants. Fungal inoculation modified the response of root hydraulic conductivity of the trembling aspen to change in the root pH. At higher pH levels, the root hydraulic conductivity of the inoculated seedlings increased thus there was an increase in aquaporin-mediated cell-to-cell water transport [66]. Growth and physiological responses of trembling aspen (*Populus tremuloides*), white spruce (*Picea glauca*) and tamarack (*Larix laricina*) seedlings were affected by the change in the root zone pH [63]. It has been shown that the change in the root zone pH would alter either AQP-mediated water transport or root hydraulic conductivity or both in diverse plant species [66]. A large and rapid change of root water flow rates and root hydraulic conductivity was observed when birch roots were exposed to different root pH values. It was suggested that the water channel function could be affected below or above an optimum pH range and result in a decrease of water flow through roots and leading to a loss of leaf hydration and stomatal closure [67]. It was recently proposed that Mono- and divalent-cations are also important in regulation of AQPs under low pH [63]. For example, when *Arabidopsis* protoplasts were treated with Ca^2+^, the decline in the cell hydraulic conductivity was observed, however, binding divalent cations to AQPs may cause changes in AQP configuration that trigger signal responses affecting the water transport under some abiotic stress conditions [68].

### Reactive oxygen species

Reactive oxygen species (ROS) control signaling responses of plants under abiotic stress conditions, however, it can cause severe oxidative damage at the tissue and cellular levels. It has been observed that the formation of ROS in parenchyma cells can be induced by high light intensity and cause reversible oxidative gating of AQPs [69, 70]. Two different regulatory mechanisms of ROS have been suggested. AQP function can be affected by oxidizing cysteine residues of the pores which lead to conformational changes of the protein and, consequently, the dephosphorylation will close the channel [9]. *OH can also attack the triple bond structures between the carbons of the plasma membrane through the oxidation of lipids and the resulting lipid radicals will close the AQP pore [70]. H_2_O_2_ can be also produced by apoplast in response to ABA and other adverse environmental conditions such as drought and salinity [71]. It has also been suggested that under stress conditions, ROS can be excessively produced and causes signal transduction to activate the AQP and close the AQP channel [9].

### Regulation of aquaporins by different chemical agents

Some chemical agents such as mercury and silver can act as inhibitors and block aquaporin transport. Mercury (often applied as HgCl_2_) is one of the most commonly used inhibitors of AQP activity [72, 73]. Most of the AQP pores are blocked by various mercurial compounds [74]. Mercurial compounds affect AQP activity by binding to the SH-groups of cysteine residues attached to the NPA motif in the central pore and blocking the pore, which leads to a disturbance of water flow [74]. It was also reported that applying sodium naphthenates decreased the hydraulic conductivity, root water transport and root respiration rate in *Populus tremuloides* [75], but the mechanisms of this inhibition are not clear. Respiration rates are often strongly linked to AQP activity, possibly through the effect on phosphorylation [72, 75–78]. A wide range of metabolic inhibitors that includes sodium azide, cycloheximide, naphthenates, mercurial compounds and other sulphydryl reagents can inhibit AQP activity. Cycloheximide, which inhibits protein synthesis, almost immediately inhibited root water flow and stomatal conductance in *Populus tremuloides* [76]. All sulphydryl reagents are considered to be AQP inhibitors since they block AQPs through oxidation of cysteine residues. Inhibition by sulphydryl reagents can be reversed by reducing agents, e.g.: 2-mercaptoethanol [72, 76, 77, 79].

### Stress response of aquaporins

Numerous studies have confirmed that the abundance of AQPs is regulated by various developmental and environmental factors including biotic and abiotic stresses. Environmental stresses such as salinity, drought and low root temperature can quickly reduce water transport rates [72, 77, 78, 80–83]. It is clear that among the different changes in environmental conditions, preserving water balance under stress conditions can be a difficult and crucial challenge for plants [84]. Therefore, plants need to have various adaptive responses to handle environmental stresses and their effects on water balance. AQPs play a key role in maintaining water homeostasis and balance under different environmental stress conditions [9, 82, 85–87]. The abundance and activity of AQPs in the plasma membrane is regulated in order to control water fluxes within the cells and in and out of the cells [1].

Abiotic stresses such as drought and salinity can alter transmembrane water movement [54, 55, 81]. Changes of AQP expression can be effectively used to regulate water transport under different abiotic stresses. However, its effectiveness can vary depending on the plant growth conditions, stages of development and tissue type as well as the duration and intensity of stress [55, 82, 86] and the type of AQP [54]. During abiotic stress, specific AQP isoforms may be expressed in certain tissues while other isoforms are expressed throughout the plant. Manipulation of AQP gene expression has greatly helped to understand plant water relations under stress conditions. Molecular analyses of regulation of the whole AQP family have often revealed complex transcriptional and posttranslational responses, with sometimes opposite patterns between the different isoforms. However, the abundance of AQP transcripts and the encoded proteins are not necessarily correlated [14, 24]. In fact, not all AQP transcripts are converted into proteins, because of the protein turn over and posttranslational modifications (PTM). PTM is a step in protein synthesis where polypeptides undergo structural and functional changes following initial synthesis of protein. Turnover is the balance between synthesis and degradation of protein [46, 88]. The exact role of AQPs in maintaining plant water status under different stress condition cannot be clearly explained because different AQP genes may be variously stimulated or reduced or may remain unchanged under abiotic stresses. In rice, AQP function was not strictly correlated with root water fluxes, but the function of AQPs became more important under drought stress conditions [89]. Stress can also affect membrane trafficking of AQPs. In Arabidopsis, salt stress was found to induce the internalization of PIP2;1 from the plasma membrane to the vacuolar lumen and this process was suppressed by the inhibitors of kinases and clathrin-mediated endocytosis [90]. Conferred stress tolerance to tobacco by SpAQP1 gene from the halophytic plant *Sesuvium portulacastrum* provides evidence for the importance of AQPs in salt stress tolerance [91].

Considering that AQPs may function in transport processes of other molecules in addition to water, understanding the nature of these complex changes may prove challenging. This complex expression patterns also suggest that water status is maintained by increased or reduced cell-to-cell water transport via AQPs under abiotic stress conditions [81].

Numerous studies have confirmed that increasing AQP expression in transgenic plants can increase plant resistance to stresses [14, 78, 92]. However, in some cases, negative effects on stress resistance have been observed in plants over-expressing heterologous AQPs [14]. This can frequently be explained by the nature and intensity of stress. In most cases, when over-expression of AQPs had beneficial effects, leaf water potentials and transpiration rates were higher compared with the wild-type plants [24, 78]. These results can be more clearly demonstrated under controlled environmental conditions. However, growth optimization through the development of stress-resistant genotypes overexpressing single AQP genes may not be effective under complex natural environmental conditions [93].

### Responses of plants to aquaporin over-expression

Physiological responses of plants to a variety of abiotic stresses can be altered by the genetic manipulation of plants (Table 1). Increasing water use efficiency and plant water retention in the cell are the important goals in the development of tolerance to abiotic stresses. The molecular mechanisms of regulation of AQP function are also important in plants over-expressing AQP genes [56]. Expression of AQP *PIP1* of *Vicia faba* (*VfPIP1*) in transgenic *Arabidopsis thaliana* improved drought resistance by the reduction of transpiration rates through stomatal closure [92]. Over-expression of gene RWC3 responsible for relative water content in transgenic lowland rice (*Oryza sativa*) showed increased resistance to polyethylene glycol (PEG) induced osmotic stress through increased root hydraulic conductivity [94]. Under salinity stress, expression of OsPIP1;1 in rice increased in leaves, but was reduced in roots [95]. Therefore, the response of each over-expressed AQP isoform towards a specific stress depends on the increase in water uptake from soil, role of AQPs in the control of water loss by transpiration and its capacity to maintain CO_2_ assimilation. The effect of interaction of a particular AQP isoform on other endogenous AQPs may alter the response to stress. The stomatal density and transpiration rates were increased in transgenic AtPIP1b-overexpressing tobacco plants, whereas they remained unchanged in transgenic NtAQP1-over-expressing *Arabidopsis* plants [96] compared to the wild-type control plants. The phenotypic changes shown by AQP over-expression play a key role in the development of tolerance during abiotic stress [92]. Stress tolerance is achieved by the cooperation between the over-expressing foreign AQPs and the endogenous AQPs. The expression of one AQP gene affects the expression patterns and distribution of endogenous AQP genes and this effect varies among plant species [97]. The expression of cucumber (*Cucumis sativus*) AQP genes CsPIP1;1 and CfPIP2;1 increased seed germination rates under high salinity. When the root temperature (RT) was lowered from 20 to 5 °C in solution culture for 30 min, *PIP2:5* over-expressing hybrid aspen *Populus tremula* × *alba* (*PtdPIP2;5ox*) AQP had significantly higher net gas exchange rates and hydraulic conductivities (L_p_) compared with the wild-type plants and recovery also was fast in transgenic lines when the RT was raised back to 20 °C [78]. When the plants were exposed to low root temperatures for 3 weeks in solution culture, gas exchange rates, water use efficiency and hydraulic conductivity of the transgenic lines were significantly higher than the wild-types but no differences in relative plant height growth and dry weight [78].

**Table 1:** Examples of attempts to improve plant resistance to abiotic stress by manipulation of AQP expression

Aquaporin gene isoform	Aquaporin over expressing plant	Promoter	Stress condition	Response	References
*VfPIP1*	*Arabidopsis thaliana*	35S	Drought (soil drying by withholding water)	Resistance	[92]
*AtPIP1;b*	*Nicotiana tabacum*	35S	Salinity (90 mM NaCl, 40 days)	Sensitive	[14]
*VvPIP2;4* *N*	*Vitis vinifera*	35S	Water stress (soil drying by withholding water, 14 days)	Sensitive	[100]
*StPIP1*	*Nicotiana tabacum*	35S rd29A	Water stress (25% PEG 6000)	Sensitive	[99]
*RWC3*	*Oryza sativa*	SWPA2	Water stress (PEG treatment, 10 h)	Resistance	[94]
*OsPIP1;1*	*Oryza sativa*	35S	Salinity (100 mM NaCl, 14 days)	Resistance	[95]
*SlTIP2;2*	*Solanum lycopersicum*	EVO205 35S	Salinity (180–200 mM NaCl)	Resistance	[93]
*HvPIP2;1*	*Oryza sativa*	35S	Salinity (100 mM NaCl, 2 weeks)	Sensitive	[88]
*TdPIP1;1*	*Nicotiana tabacum*	–	Salinity (250 mM NaCl, 30 days) Water stress (300 mM mannitol, 30 days)	Resistance Resistance	[101]
*BnPIP1*	*Nicotiana tabacum*	35S	Drought (stop irrigation, 20% PEG8000)	Resistance	[88]
*TaAQP7*	*Nicotiana tabacum*	35S	Drought (water deprivation, 20 days) Osmotic stress (150–300 mM mannitol, 7–12 days)	Resistance Resistance	[102]
*AtPIP1;4, AtPIP2;5*	*Arabidopsis thaliana, Nicotiana tabacum*	35S	Water stress (100–400 mM mannitol 12–24 h, withholding water 15 days) Salinity (50 mM NaCl 14 days) Cold (10 °C, 24 h)	No effect	[97]

The expression of leaf gourd (*Cucurbita ficifolia*) AQP gene *CfPIP2;1* increased the survival rate of *Arabidopsis* plants under extreme drought [98]. The functions of *PIP2* subfamily AQPs will be more affected than the *PIP1* subfamily, under severe abiotic stress [98]. It is not clear how the foreign AQP gene modifies the function of endogenous PIPs *of Arabidopsis.* As mentioned above, over-expression of plant AQPs did not always improve tolerance to abiotic stress [14, 99, 100]. Over-expression of AQP gene *AtPIP1b* in *Arabidopsis* increased the growth rate, gas exchange and stomatal density in *Nicotiana tabacum* under favorable conditions, but decreased the growth, under drought stress conditions [14].

## Conclusions

During the last years, intensive research has focused on understanding the structure and function of plant AQPs and the role of plant AQPs in water transport and stress responses. These studies have helped to improve the understanding of the functions of different AQP isoforms in water and nutrient uptake as well as regulation of leaf and root hydraulics, plant growth and development, and stress response. Progress has been made in understanding the molecular bases of AQP transport selectivity and gating. Studies at the molecular level and gene expression analyses using methods such as RT-PCR or different expression systems, suggest different co-translation and post-translational mechanisms and molecular interactions between AQP isoforms and regulatory proteins. Signaling events and environmental changes can affect the AQP function and regulation. AQPs can also play an important role in plant growth and controlling tissue expansion. However, manipulations of AQP gene expression for the improvement of plant stress resistance remain to be a challenge.

## References

[1] Maurel C (2007). Plant aquaporins: novel functions and regulation properties. FEBS Lett.

[2] Chrispeels MJ, Agre P (1994). Aquaporins: water channel proteins of plant and animal cells. Trends Biochem Sci.

[3] Bramley H, Turner DW, Tyerman SD, Turner NC (2007). Water flow in the roots of crop species: the influence of root structure, aquaporin activity, and water logging. Adv Agron.

[4] Gomes D, Agasse A, Thiebaud P, Delrot S, Geros H, Chaumont F (2009). Aquaporins are multifunctional water and solute transporters highly divergent in living organisms. Biochim Biophys Acta..

[5] Zwiazek JJ, Tan X, Xu H, Navarro-Ródenas A, Morte A (2017). Functional significance of oxygen transport through aquaporins. Sci Rep.

[6] Murata K, Mitsuoka K, Hirai T, Walz T, Agre P, Heymann JB, Engel A, Fujiyoshi Y (2000). Structural determinants of water permeation through aquaporin-1. Nature.

[7] Chaumont F, Moshelion M, Daniels MJ (2005). Regulation of plant aquaporin activity. Biol Cell.

[8] Zardoya R (2005). Phylogeny and evolution of the major intrinsic protein family. Biol Cell.

[9] Luu DT, Maurel C (2005). Aquaporins in a challenging environment: molecular gears for adjusting plant water status. Plant Cell Environ.

[10] Clarkson DT, Carvajal M, Henzler T, Waterhouse RN, Smyth AJ, Cooke DT, Steudle E (2000). Root hydraulic conductance: diurnal aquaporin expression and the effects of nutrient stress. J Exp Bot.

[11] Maurel C, Chrispeels MJ (2001). Aquaporins. A molecular entry into plant water relations. Plant Physiol.

[12] Javot H, Lauvergeat V, Santoni V, Martin-Laurent F, Guclu J, Vinh J, Heyes J, Franck KI, Schaffner AR, Bouchez D, Maurel C (2003). Role of a single aquaporin isoform in root water uptake. Plant Cell.

[13] Postaire O, Tournaire-Roux C, Grondin A, Boursiac Y, Morillon R, Schaffner AR, Maurel C (2010). A PIP1 aquaporin contributes to hydrostatic pressure-induced water transport in both the root and rosette of *Arabidopsis*. Plant Physiol.

[14] Aharon R, Shahak Y, Wininger S, Bendov R, Kapulnik Y, Galili G (2003). Overexpression of a plasma membrane aquaporin in transgenic tobacco improves plant vigour under favourable growth conditions but not under drought or salt stress. Plant Cell.

[15] Wudick MW, Luu D-T, Maurel C (2009). A look inside: localization patterns and functions of intracellular plant aquaporins. New Phytol.

[16] Frangne N, Maeshima M, Schäffner AR, Mandel T, Martinola E, Bonnemain JL (2001). Expression and distribution of a vacuolar aquaporin in young and mature leaf tissues of *Brassica napus* in relation to water fluxes. Planta.

[17] Danielson JAH, Johanson U (2008). Unexpected complexity of the aquaporin gene family in the moss *Physcomitrella patens*. BMC Plant Biol.

[18] Maurel C, Verdoucq L, Luu DT, Santoni V (2008). Plant aquaporins: membrane channels with multiple integrated functions. Annu Rev Plant Biol.

[19] Hussain SS, Iqbal MT, Arif MA, Amjad M (2011). Beyond osmolytes and transcription factors: drought tolerance in plants via protective proteins and aquaporins. Biol Plant.

[20] Marjanović Z, Uehlein N, Kaldenhoff R, Weiss M, Hamp R, Zwiazek JJ, Nehls U (2005). Aquaporins in poplar: what a difference a symbiont makes!. Planta..

[21] Tuskan GA, DiFazio S, Jansson S, Bohlmann J (2006). The genome of black cottonwood, *Populus trichocarpa* (Torr. & Gray). Science.

[22] Gupta AB, Sankararamakrishnan R (2009). Genome-wide analysis of major intrinsic proteins in the tree plant *Populus trichocarpa*: characterization of XIP subfamily of aquaporins from evolutionary perspective. BMC Plant Biol.

[23] Park W, Scheffler BE, Bauer PJ, Campbell BT (2010). Identification of the family of aquaporin genes and their expression in upland cotton (*Gossypium hirsutum* L.). BMC Plant Biol..

[24] Almeida-Rodriguez AM, Cooke JEK, Yeh F, Zwiazek JJ (2010). Functional characterization of drought- responsive aquaporins in *Populus balsamifera* and *Populus simonii* × *balsamifera* clones with different drought resistance strategies. Physiol Plant.

[25] Kaldenhoff R, Fisher M (2006). Aquaporins in plants. Acta Physiol..

[26] Wayne R, Tazawa M (2010). Nature of the water channels in the internodal cells of *Nitellopsis*. J Membr Biol..

[27] Chaumont F, Barrieu F, Jung R, Chrispeels MJ (2000). Plasma membrane intrinsic proteins from maize cluster in two sequence subgroups with differential aquaporin activity. Plant Physiol.

[28] Schuurmans JA, van Dongen JT, Rutjens BP, Boonman A, Pieterse CM, Borstlap AC (2003). Members of the aquaporin family in the developing pea seed coat include representative of the PIP, TIP, and NIP subfamilies. Plant Mol Biol.

[29] Hanba YT, Shibasaka M, Hayashi Y, Hayakawa T, Kasamo K, Terashima I, Katsuhara M (2004). Overexpression of the barley aquaporin HvPIP2; 1 increases internal CO_2_ conductance and CO_2_ assimilation in the leaves of transgenic rice plants. Plant Cell Physiol.

[30] Siefritz F, Biela A, Eckert M, Otto B, Uehlein N, Kaldenhoff R (2001). The tobacco plasma membrane aquaporin NtAQP1. J Exp Bot.

[31] Li G, Santoni V, Maurel C (2014). Plant aquaporins: roles in plant physiology. Biochem Biophys Acta.

[32] Johnson KD, Höfte H, Chrispeels MJ (1990). An intrinsic tonoplast protein of protein storage vacuoles in seeds is structurally related to a bacterial solute transporter (GIpF). Plant Cell.

[33] Delrot S, Picaud S, Gaudillère JP, Delrot S, Picaud S, Gaudillère JP, Roubelakis-Angelakis KA (2001). Water transport and aquaporins in grapevine. Molecular biology and biotechnology of grapevine.

[34] Fleurat-Lessard P, Michonneau P, Maeshima M, Drevon JJ, Serraj R (2005). The distribution of aquaporin subtypes (PIP1, PIP2 and γ-TIP) is tissue dependent in soybean (*Glycine max*) root nodules. Ann Bot.

[35] Nozaki K, Ishii D, Ishibashi K (2008). Intracellular aquaporins: clues for intracellular water transport?. Eur J Physiol.

[36] Kirch HH, Vera-Estrella R, Golldack D, Quigley F, Michalowski CB, Barkla BJ, Bohnert HJ (2000). Expression of water channel proteins in *Mesembryanthemum crystallinum*. Plant Physiol.

[37] Fortin MG, Zelechowska M, Verma DPS (1985). Specific targeting of membrane nodulins to the bacteroid-enclosing compartment in soybean nodules. EMBO J..

[38] Weig A, Deswarte C, Chrispeels MJ (1997). The major intrinsic protein family of *Arabidopsis* has 23 members that form three distinct groups with functional aquaporins in each group. Plant Physiol.

[39] Noronha H, Araújo D, Conde C, Martins AP, Soveral G, Chaumont F, Delrot S, Gerós H (2016). The grapevine uncharacterized intrinsic protein 1 (VvXIP1) is regulated by drought stress and transports glycerol, hydrogen peroxide, heavy metals but not water. PLoS ONE.

[40] Liang WH, Li L, Zhang F, Liu YX, Li MM, Shi HH, Yang XG (2011). Effects of abiotic stress, light, phytochromes and phytohormones on the expression of OsAQP, a rice aquaporin gene. Plant Growth Regul.

[41] Lin W, Peng Y, Li G, Arora R, Tang Z, Su W, Cai W (2007). Isolation and functional characterization of PgrTIPI, a hormone-autotrophic cells-specific tonoplast aquaporin in ginseng. J Exp Bot.

[42] Bae EK, Lee H, Lee JS, Noh EW (2011). Drought, salt and wounding stress induce the expression of the plasma membrane intrinsic protein 1 gene in poplar (*Populus alba* x *P. tremula* var. *glandulosa*). Gene.

[43] Bustin SA, Benes V, Garson JA, Hellemans J, Huggett J, Kubista M, Mueller R, Nolan T, Pfaffl MW, Shipley GL, Vandesompele J, Wittwer CT (2009). The MIQE guidelines: minimum information for publication of quantitative real-time PCR experiments. Clin Chem.

[44] Hachez C, Zelazny EF (2006). Chaumont modulating the expression of aquaporin genes in planta: a key to understand their physiological functions?. Biochem Biophys Acta.

[45] Martre P, Morillon R, Barrieu F, North G, Nobel P, Chrispeels M (2002). Plasma membrane aquaporin play a significant role during recovery from water deficit. Plant Physiol.

[46] Aroca R, Amodeo G, Fernándezllescas S, Herman EM, Chaumont F, Chrispeels MJ (2005). The role of aquaporins and membrane damage in chilling and hydrogen peroxide induced changes in the hydraulic conductance of maize roots. Plant Physiol.

[47] Verdoucq L, Grondin A, Maurel C (2008). Structure–function analysis of plant aquaporin *At*PIP2;1 gating by divalent cations and protons. Biochem J.

[48] Tornroth-Horsefield S, Wang Y, Hedfalk K, Johanson U, Karlsson M, Tajkhorshid E, Neutze R, Kjellbom P (2006). Structural mechanism of plant aquaporin gating. Nature.

[49] Azad AK, Sawa Y, Ishikawa T, Shibata H (2004). Characterization of protein phosphatase 2A acting on phosphorylated plasma membrane aquaporin of tulip petals. Biosci Biotechnol Biochem.

[50] Fetter K, Van Wilder V, Moshelion M, Chaumont F (2004). Interactions between plasma membrane aquaporins modulate their water channel activity. Plant Cell.

[51] Zelazny E, Borst JW, Muylaert M, Batoko H, Hemminga M, Chaumont F (2007). FRET Imaging in living maize cells reveals that plasma membrane aquaporins interact to regulate their subcellular localization. Proc Natl Acad Sci USA.

[52] Johansson I, Karlsson M, Shukla V, Chrispeels M, Larsson C, Kjellbom P (1998). Water transport activity of the plasma membrane aquaporin PM28A is regulated by phosphorylation. Plant Cell.

[53] Bots M, Feron R, Uehlein N, Weterings K, Kaldenhoff R, Mariani T (2005). PIP1 and PIP2 aquaporins are differently expressed during tobacco anther and stigma development. J Exp Bot.

[54] Jang JY, Kim DG, Kim YO, Kim JS, Kang H (2004). An expression analysis of a gene family encoding plasma membrane aquaporins in response to abiotic stresses in *Arabidopsis thaliana*. Plant Mol Biol.

[55] Alexandersson E, Fraysse L, Sjovall-Larsen S, Gustavsson S, Fellert M, Karlsson M, Johanson U, Kjellbom P (2005). Whole gene family expression and drought stress regulation of aquaporins. Plant Mol Biol.

[56] Zhu C, Schraut D, Hartung W, Schaffner AR (2005). Differential responses of maize MIP genes to salt stress and ABA. J Exp Bot.

[57] Wan X, Zwiazek JJ (2001). Root water flow and leaf stomatal conductance in aspen (*Populus tremuloides*) seedlings treated with abscisic acid. Planta.

[58] Beaudette PC, Chlup M, Yee J, Emery RN (2007). Relationships of root and aquaporin gene expression in *Pisum sativum*: diurnal patterns and the response to HgCl_2_ and ABA. J Exp Bot.

[59] Kamaluddin M, Zwiazek JJ (2002). Naphthenic acids inhibit root water transport, gas exchange and leaf growth in aspen (*Populus tremuloides*) seedlings. Tree Physiol.

[60] Kamaluddin M, Zwiazek JJ (2002). Ethylene enhances water transport in hypoxic aspen. Plant Physiol.

[61] Tournaire-Roux C, Sutka M, Javot H, Gou E, Gerbeau P, Luu DT, Maurel C (2003). Cytosolic pH regulates root water transport during anoxic stress through gating of aquaporins. Nature.

[62] Zhao CX, Shao HB, Chu LY (2008). Aquaporin structure–function relationships: water flow through plant living cells. Colloids Surf B.

[63] Zhang W, Calvo-Polanco M, Chen ZC, Zwiazek JJ (2013). Growth and physiological responses of trembling aspen (*Populus tremuloides*), white spruce (*Picea glauca*) and tamarack (*Larix laricina*) seedlings to root zone pH. Plant Soil.

[64] Sade N, Gebremedhin A, Moshelion M (2012). Risk-taking plants: anisohydric behavior as a stress-resistance trait. Plant Signal Behav..

[65] Yaneff A, Sigaut L, Gómez N, Fandiño CA, Alleva K, Pietrasanta LI, Amodeo G (2016). Loop B serine of a plasma membrane aquaporin type PIP2 but not PIP1 plays a key role in pH sensing. Biochim Biophys Acta..

[66] Siemens JA, Zwiazek JJ (2011). *Hebeloma crustuliniforme* modifies root hydraulic responses of trembling aspen (*Populus tremuloides*) seedlings to changes in external pH. Plant Soil.

[67] Kamaluddin M, Zwiazek JJ (2004). Effects of root medium pH on water transport in paper birch (*Betula papyrifera*) seedlings in relation to root temperature and abscisic acid treatments. Tree Physiol.

[68] Gerbeau P, Amodeo G, Henzler T, Santoni V, Ripoche P, Maurel C (2002). The water permeability of *Arabidopsis* plasma membrane is regulated by divalent cations and pH. Plant J..

[69] Kim YX, Steudle E (2009). Gating of aquaporins by light and reactive oxygen species in leaf parenchyma cells of the midrib of *Zea mays*. J Exp Bot.

[70] Henzler T, Ye Q, Steudle E (2004). Oxidative gating of water channels (aquaporins) in *Chara* by hydroxyl radicals. Plant Cell Environ.

[71] Jubany-Marí T, Munné-Bosch S, Lopez-Carbonell M, Alegre L (2009). Hydrogen peroxide is involved in the acclimation of the Mediterranean shrub, *Cistus albidus* L., to summer drought. J Exp Bot.

[72] Wan X, Zwiazek JJ (1999). Mercuric chloride effects on root water transport in aspen seedlings. Plant Physiol.

[73] Niemietz CM, Tyerman SD (2002). New potent inhibitors of aquaporins: silver and gold compounds inhibit aquaporins of plant and human origin. FEBS Lett.

[74] Quigley F, Rosenberg J, Shachar-Hill Y, Bohnert H (2001). From genome to function: the *Arabidopsis* aquaporins. Genome Biol.

[75] Kamaluddin M, Zwiazek JJ (2001). Metabolic inhibition of root water flow in red-osier dogwood (*Cornus stolonifera*) seedlings. J Exp Bot.

[76] Voicu MC, Zwiazek JJ (2004). Cycloheximide inhibits root water flow and stomatal conductance in aspen (*Populus tremuloides*) seedlings. Plant Cell Environ.

[77] Lee SH, Zwiazek JJ, Chung GC (2008). Light induced transpiration alters cell water relations in figleaf gourd (*Cucurbita ficifolia*) seedlings exposed to low root temperatures. Physiol Plant.

[78] Ranganathan R, Kayal WE, Cooke JEK, Janusz JJ (2016). Responses of hybrid aspen over-expressing a PIP2;5 aquaporin to low root temperature. J Plant Physiol..

[79] Tyerman SD, Bohnert HJ, Maurel C, Steudle E, Smith JA (1999). Plant aquaporins: their molecular biology, biophysics and significance for plant water relations. J Exp Bot.

[80] Wan X, Landhäusser SM, Zwiazek JJ, Lieffers VJ (1999). Root water flow and growth of aspen (*Populus tremuloides*) at low root temperatures. Tree Physiol.

[81] Javot H, Maurel C (2002). The role of aquaporins in root water uptake. Ann Bot.

[82] Siemens JA, Zwiazek JJ (2003). Effects of water deficit stress and recovery on the root water relations of trembling aspen (*Populus tremuloides*) seedlings. Plant Sci.

[83] Lee SH, Chung GC, Jang JY, Ahn SJ, Zwiazek JJ (2012). Over-expression of PIP2;5 aquaporin alleviates effects of low root temperature on cell hydraulic conductivity and growth in *Arabidopsis*. Plant Physiol.

[84] Mahdieh M, Mostajeran A, Horie T, Katsuhara M (2008). Drought stress alters water relations and expression of PIP-type aquaporin genes in *Nicotiana tabacum* plants. Plant Cell Physiol.

[85] Wan X, Zwiazek JJ, Lieffers VJ, Landhäusser SM (2001). Hydraulic conductance in aspen (*Populus tremuloides*) seedlings exposed to low root temperatures. Tree Physiol.

[86] Galmes J, Pou A, Alsina MM, Tomas M, Medrano H, Flexas J (2007). Aquaporin expression in response to different water stress intensities and recovery in Richter-110 *(Vitis* sp.): relationship with ecophysiological status. Planta.

[87] Ranganathan K, Walid EK, Cooke JEK, Equiza MA, Vaziriyeganeh M, Zwiazek JJ (2017). Over-expression of PIP2;5 aquaporin alleviates gas exchange and growth inhibition in poplars exposed to mild osmotic stress with polyethylene glycol. Acta Physiol Plant.

[88] Yu Q, Hu Y, Li J, Wu Q, Lin Z (2005). Sense and antisense expression of plasma membrane aquaporin BnPIP1 from *Brassica napus* in tobacco and its effects on plant drought resistance. Plant Sci.

[89] Grondin A, Mauleon R, Vadez V, Henry A (2016). Root aquaporins contribute to whole plant water fluxes under drought stress in rice (*Oryza sativa* L.). Plant Cell Environ.

[90] Ueda M, Tsutsumi N, Fujimoto M (2016). Salt stress induces internalization of plasma membrane aquaporin into the vacuole in *Arabidopsis thaliana*. Biochem Biophys Res Commun.

[91] Chang W, Liu X, Zhu J, Fan W, Zhang Z (2016). An aquaporin gene from halophyte *Sesuvium portulacastrum*, SpAQP1, increases salt tolerance in transgenic tobacco. Plant Cell Rep.

[92] Cui XH, Hao FS, Chen H, Chen J, Wang XC (2008). Expression of the *Vicia faba VfPIP1* gene in *Arabidopsis thaliana* plants improves their drought resistance. J Plant Res.

[93] Sade N, Vinocur BJ, Diber A, Shatil A, Ronen G, Nissan H, Moshelion M (2009). Improving plant stress tolerance and yield production: is the tonoplast aquaporin SlTIP2; 2 a key to isohydric to anisohydric conversion. New Phytol.

[94] Lian HL, Yu X, Ye Q, Ding X, Kitagawa Y, Kwak SS, Su WA, Tang ZC (2004). The role of aquaporin RWC3 in drought avoidance in rice. Plant Cell Physiol.

[95] Liu C, Fukumoto T, Matsumoto T, Gena P, Frascaria D, Kaneko T, Katsuhara M, Kitagawa Y (2013). Aquaporin *OsPIP1;1* promotes rice salt resistance and seed germination. Plant Physiol Biochem.

[96] Cui X, Hao F, Chen H, Cai J, Chen J, Wang X (2005). Isolation and expression of an aquaporin-like gene *VfPIP1* in *Vicia faba*. Prog Nat Sci.

[97] Jang JY, Lee SH, Rhee JY, Chung GC, Ahn SJ, Kang H (2007). Transgenic *Arabidopsis* and tobacco plants overexpressing an aquaporin respond differently to various abiotic stresses. Plant Mol Biol.

[98] Jang JY, Rhee JY, Kim DG, Chung GC, Lee JH, Kang H (2007). Ectopic expression of a foreign aquaporin disrupts the natural expression patterns of endogenous aquaporin genes and alters plant responses to different stress conditions. Plant Cell Physiol.

[99] Wu WZ, Peng XL, Wang D (2009). Isolation of a plasmalemma aquaporin encoding gene StPIP1 from *Solanum tuberosum* L. and its expression in transgenic tobacco. Agric Sci China..

[100] Perrone I, Gambino G, Chitarra W, Vitali M, Pagliarani C, Riccomagno N, Balestrini R, Lovisolo C (2012). The grapevine root-specific aquaporin VvPIP2;4N controls root hydraulic conductance and leaf gas exchange under well-watered conditions but not under water stress. Plant Physiol.

[101] Ayadi M, Cavez D, Miled N, Chaumont F, Masmoud K (2011). Identification and characterization of two plasma membrane aquaporins in durum wheat (*Triticum turgidum* L. subsp. *durum*) and their role in abiotic stress tolerance. Plant Physiol Biochem.

[102] Zhou S, Hu W, Deng X, Ma Z, Chen L, Huang C, Wang C, Wang J, He Y, Yang G, He G (2012). Overexpression of the wheat aquaporin gene, *TaAQP7*, enhances drought tolerance in transgenic tobacco. PLoS ONE.

